# Bacterial Culture-Negative Acute Infective Endocarditis With Vegetation in Native Bicuspid Aortic Valve

**DOI:** 10.7759/cureus.35101

**Published:** 2023-02-17

**Authors:** Michael Sunnaa, Ritika Dhawan, Deborah Tabachnick, Steve Attanasio

**Affiliations:** 1 Internal Medicine, Rush University Medical Center, Chicago, USA; 2 Cardiovascular and Thoracic Surgery, Rush University Medical Center, Chicago, USA; 3 Cardiovascular Medicine, Rush University Medical Center, Chicago, USA

**Keywords:** blood culture negative endocarditis, culture negative, valvular vegetation, infectious endocarditis, valve, aortic, biscuspid

## Abstract

A 51-year-old male with a past medical history of bicuspid aortic valve, hypertension, and anterior cerebral artery stroke of unclear etiology three months earlier, presented to the Emergency Department with progressive shortness of breath, hemoptysis, and night sweats. The patient's echocardiogram revealed a mobile mass greater than 1.0 cm in diameter on the bicuspid aortic valve, which was not present on the echocardiogram three months prior, during his stroke workup. Per modified Duke's criteria, this patient was found to have 'definite' infective endocarditis despite persistently negative blood cultures. The patient underwent urgent surgical aortic valve replacement and a ventricular septal defect was noted that was not seen on prior imaging. The patient was discharged on intravenous antibiotics and warfarin. The patient was able to return to his normal functional status weeks after surgery, and is continuing to exercise without limitation. This case provides an example of patients with bicuspid aortic valves having an increased propensity for developing infective endocarditis. While also highlighting the interesting intra-operative images and presentation of acute culture-negative endocarditis with vegetation, and the subsequent repair, treatment, and recovery.

## Introduction

Bicuspid aortic valve (BAV) affects 1-2% of the population and is the most common congenital cardiac malformation [1]. BAVs can be familial, with 20-30% of those with bicuspid aortic valves having a first-degree relative with a bicuspid aortic valve [2]. BAVs are also noted to have a male predominance with a male-female ratio of 3:1 [3]. A retrospective cohort study in 2012 demonstrated that BAVs are more common in Caucasian Americans than in African Americans [4]. Bicuspid aortic valves have a higher incidence of infective endocarditis (IE). Adverse events of IE are heart failure, peripheral embolism, embolic stroke, persistent bacteremia, and intracardiac complications [5,6]. The magnitude of incidence of IE is a matter of conflicting research, with various sources stating that the incidence ranges from 5% to 30% [7,8]. Of note, there is an association between ventricular septal defects (VSD) and BAV; perturbations in the endocardial cushion remodeling process have been associated with the development of both BAV and VSD [9]. 

## Case presentation

A 51-year-old male with a past medical history of bicuspid aortic valve, hypertension, and anterior cerebral artery stroke of unclear etiology three months earlier, presented to the Emergency Department with progressive shortness of breath of two days duration. Additional symptoms included night sweats, exertional fatigue, and hemoptysis of one-day duration. The patient did not have a history of intravenous drug use, have indwelling catheters, and was not on hemodialysis. 

**Figure 1:**
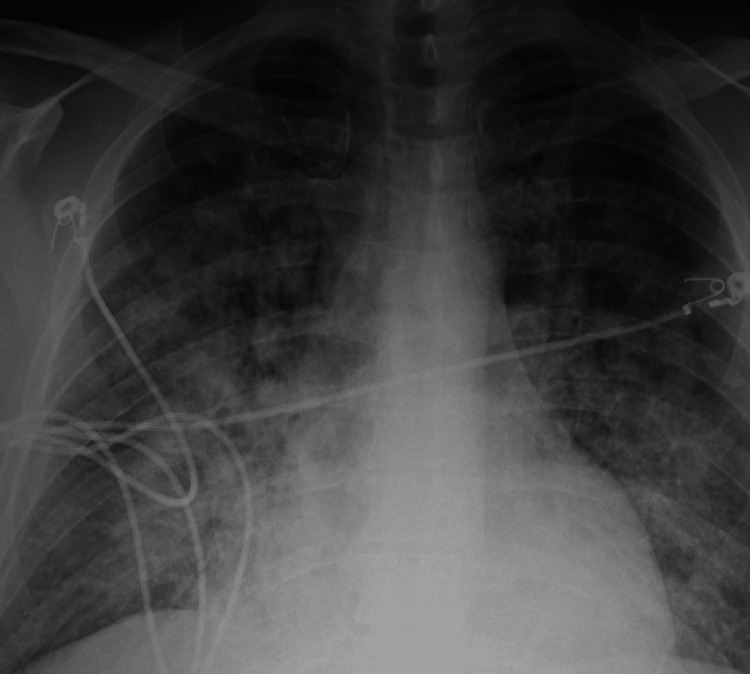
Portable chest x-ray revealing acute pulmonary edema

**Figure 2:**
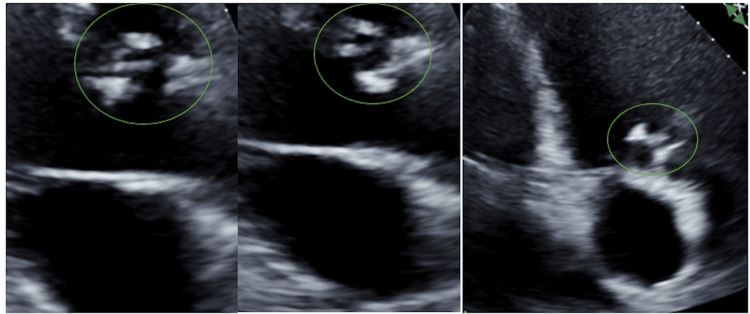
Echocardiogram images illustrating vegetation on the aortic valve (circled)

On presentation, the patient was afebrile, with blood pressure of 78/41 millimeters of mercury, heart rate of 106 beats per minute, and oxygen saturation of 79%. Chest X-ray revealed acute pulmonary edema (Figure 1). The patient's pulmonary edema was treated with diuresis and non-invasive positive pressure ventilation. Blood cultures were obtained prior to the initiation of empirical antibiotic therapy. Electrocardiogram and troponin levels were unremarkable, and a transthoracic echocardiogram demonstrated a left ventricular ejection fraction of 60-65%, hypokinesis of the apical lateral and apical anterior and mid anterior myocardium, a bicuspid aortic valve with moderate regurgitation, and a mobile mass greater than 1.0 cm in diameter (Figure 2). Presurgical coronary angiography showed no evidence of coronary artery disease. Of note, the workup of the stroke three months prior to presentation did not show vegetation of the aortic valve, ventricular septal defect, or a patent foramen ovale on transesophageal echocardiogram. 

**Figure 3:**
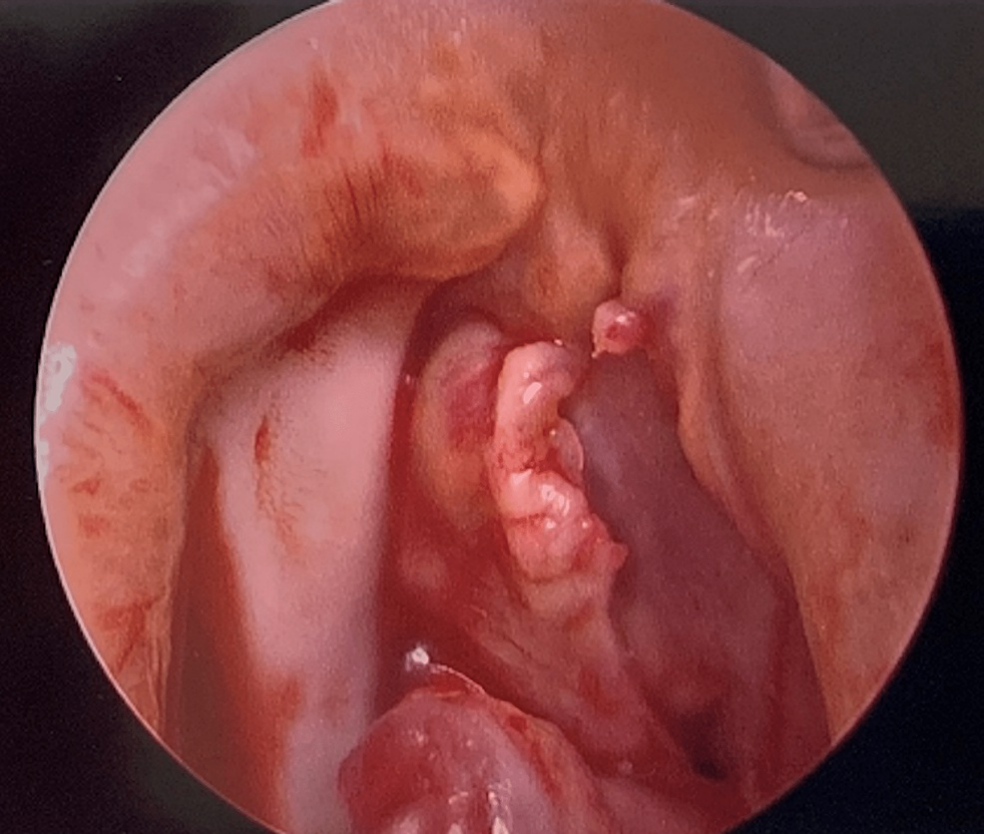
Vegetation and destruction of aortic leaflets

**Figure 4:**
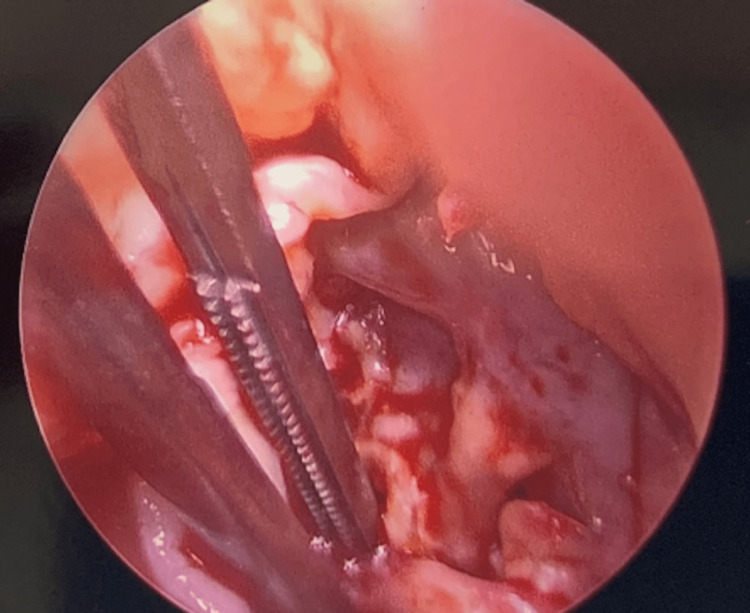
Illustration of ventricular septal defect

**Figure 5:**
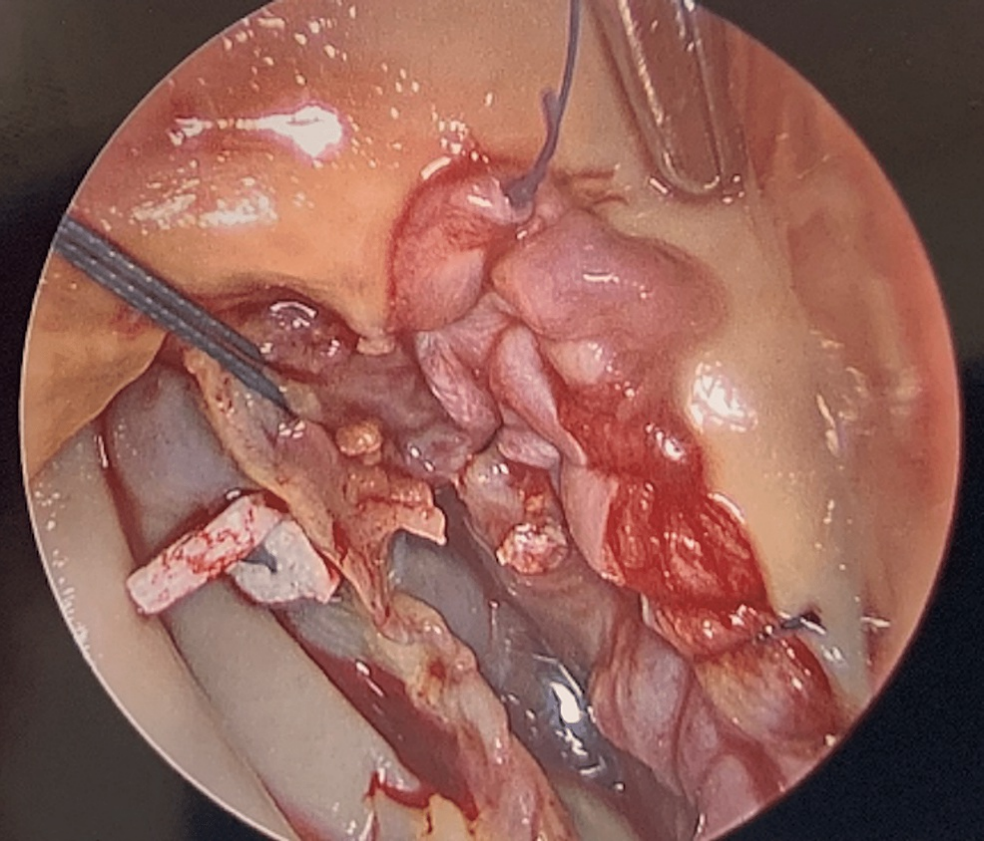
Patch repair of ventricular septal defect

The patient underwent urgent aortic valve replacement and vegetation and destruction of aortic leaflets were discovered intra-operatively (Figure 3). In surgery, he was found to have a ventricular septal defect from the left ventricular outflow tract extending to the right ventricular outflow tract (Figure 4). Surgery included the closure of VSD at the nadir of the right coronary cusp using an autologous pericardial patch (Figure 5) and aortic valve replacement with a 25mm St. Jude mechanical valve. Blood cultures were persistently negative and pathology from surgery showed a 1.2 x 0.8 x 0.3 cm roughened calcified yellow-tan tissue fragment. 

The postoperative period was uneventful. Patient was discharged on intravenous vancomycin and ceftriaxone for 42 days and warfarin with close follow up. At present, he is not experiencing chest discomfort or shortness of breath. He is now able to exercise at the gym three days a week and has no exertional chest pain or dyspnea.

## Discussion

Given the patient’s recent ischemic stroke of unknown origin, the vegetation of the aortic valve could have been the source. Despite not being present on earlier imaging, this case suggests quick propagation of the vegetation instead of a false negative on previous cardiac imaging. However, given the acute nature of symptoms, this is more likely acute IE, which propagates more so in days to weeks rather than months [10]. Furthermore, subacute infectious endocarditis has less of a propensity to hematogenously seeded extra-cardiac sites [10]. Broadly, the sensitivity of discovering vegetation on transthoracic echocardiography is 75%, and 85-90% on transesophageal echocardiography [11]. The patient was deemed 'definite' in having IE by Modified Duke's Criteria by the following: pathological criteria, evidence of endocardial involvement, fever, and pre-existing heart condition [12].

While this patient’s embolic event preceded the presence of a detected large vegetation (defined as greater than 10mm), we know that large vegetations in left-sided endocarditis are a predictor of embolic events [10]. In addition, studies and meta-analyses have shown increased mortality from large vegetations compared to medium (5-9mm) or small (<5 mm) vegetations [13,14]. This case also brings to attention the prevalence of blood culture-negative endocarditis (BCNE). Common culprits of BCNE include organisms such as *Coxiella* and *Bartonella*; however, 16s ribosomal RNA testing was not done to fully identify the organism. BCNE accounts for up to 20% of IE [15]. While the most common cause of BCNE remains the initiation of antibiotics prior to culture, our case represents a true case of culture-negative endocarditis. 

## Conclusions

This case highlights both unique intraoperative and echocardiogram findings in a patient with IE in a bicuspid aortic valve. The treatment plan of cardiac surgery with replacement of valve with a mechanical valve with discharge on warfarin has been successful thus far at the time of writing this case.
